# Unfavorable fractures during Sagittal Split Osteotomy. What are the Risk Factors? A Retrospective Tomographic Study

**DOI:** 10.4317/jced.62726

**Published:** 2025-05-01

**Authors:** Thalles Moreira Suassuna, Elenisa Glaucia Ferreira dos Santos, Sérgio Murilo Cordeiro de Melo Filho, Maria Taywri Almeida Costa, José Rodrigues Laureano Filho, Fábio Andrey da Costa Araújo

**Affiliations:** 1 Doutorando em Cirurgia e Traumatologia Buco-Maxilo-Facial na Faculdade de Odontologia de Pernambuco - Universidade de Pernambuco UPE; 2 Mestranda em Cirurgia e Traumatologia Buco-Maxilo-Facial na Faculdade de Odontologia de Pernambuco - Universidade de Pernambuco UPE; 3 Residente de Cirurgia Buco-Maxilo-Facial do Hospital Universitário Oswaldo Cruz - Universidade de Pernambuco UPE; 4 Graduanda em odontologia pela universidade da Amazônia UNAMA; 5 Professor associado de Cirurgia e Traumatologia Buco-Maxilo-Facial na Faculdade de Odontologia de Pernambuco - Universidade de Pernambuco UPE; 6 Professor adjunto de Cirurgia e Traumatologia Buco-Maxilo-Facial na Faculdade de Odontologia de Pernambuco - Universidade de Pernambuco UPE

## Abstract

**Background:**

This study aims to analyze the epidemiology of “Bad Split” (BS) during Sagittal Osteotomy of the Mandible, identifying anatomical and technical risk factors associated with its occurrence.

**Material and Methods:**

A retrospective analysis was conducted on 157 patients (314 osteotomies) over five years. Multi-slice helical CT scans, both pre- and post-operative, were examined to classify BS and identify potential risk factors. Anatomical variables included the presence of third molars, edentulism, prior fixation systems, mandibular ramus dimensions, lingula position, and alveolar crest height. Technical aspects of osteotomy execution were also assessed. Measurements were performed using Dolphin Imaging Software 11.95 after orienting the skull in the Natural Head Position.

**Results:**

The incidence of BS was 3.2% (10 patients), with a slight left-side predominance (60%). Class A BS (distal fracture of the proximal segment) was the most common (60%). Anatomically, 83.3% of Class A cases had a more anteriorly positioned lingula and 66% presented a lower alveolar crest. Technically, 80% of BS cases showed deviations in osteotomy execution, with incomplete osteotomy at the basal level being the most frequent (60%).

**Conclusions:**

This study suggests that technical factors, particularly osteotomy execution, play a more decisive role in BS occurrence than anatomical variables. Surgical precision is crucial, emphasizing careful osteotomy techniques to minimize the risk of BS, especially in anatomically predisposed mandibles.

** Key words:**Bad split, Intraoperative complications, Bilateral sagittal split osteotomy, Orthognathic surgery.

## Introduction

Unfavorable fracture, or “Bad Split” (BS), during Sagittal Osteotomy of the Mandible remains one of the primary complications associated with this technique, from its initial description and the present day (1-3).The incidence can reach up to 15%.The occurrence of this complication can make the procedure significantly more challenging, time-consuming, and morbid for the patient, leading to instability, infection, and malunion. Several factors have been identified that may increase the risk of BS. These factors can relate to the patient, such as age, anatomy, and the presence of teeth in the region, or to the execution of the procedure itself. However, the literature is not yet conclusive about which factors are most determining and lacks tomographic studies that evaluate anatomical parameters and the execution of the osteotomy. To epidemiologically study the patterns of Bad Split and identify risk factors that may contribute to its occurrence (4-6).

## Material and Methods

This was a retrospective study involving 157 patients (314 osteotomies) operated on over five years by a hospital’s surgical team, using the classic Sagittal Osteotomy popularized by Traunner and Obwegeser, later modified by Dal Pont and revised by Epker. The sample comprised patients diagnosed with intraoperative unfavorable fractures, necessitating additional osteotomies for appropriate separation between the proximal and distal segments. Multi-slice helical CT scans, both pre- and post-operatively, were studied to classify BS and identify possible risk factors. The analyzed criteria included: presence of third molars; edentulism; presence of prior fixation systems; thickness, height, and length of the ramus; anteroposterior location of the lingula; and height of the alveolar crest. Comparisons were always made with the contralateral side of the same patient. The executed osteotomies were also analyzed to assess whether there were differences compared to the opposite side, deviation, or incomplete cuts. The examinations were analyzed using Dolphin Imaging Software 11.95, where, after orienting the skull in the Natural Head Position, measurements were performed using the “Digitize-Measure - 2D line” tool. The research was conducted in accordance with the Code of Ethics of the World Medical Association (Declaration of Helsinki) and was approved by the Ethics Committee, under the umbrella project CAAE 74613823.2.0000.5207. It posed no risk to participants, as the intervention had already been performed and no additional examinations beyond routine were required.

## Results

**Figure 1:**
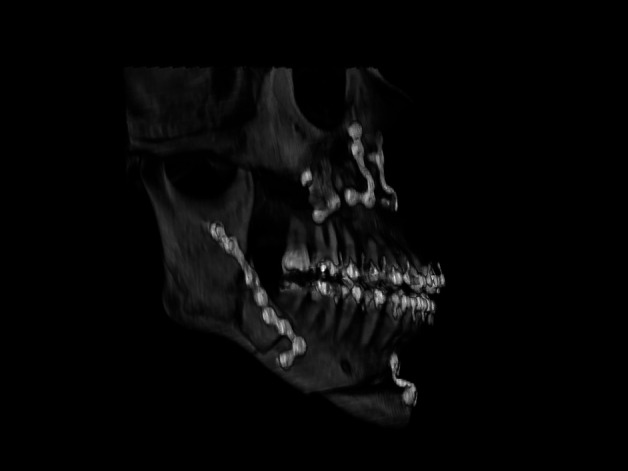
Type A of badsplit. The most common type found in our study, accounting for 60% of cases.

Ultimately, 10 patients (6.4%) with intraoperative diagnosis of unilateral BS were included, representing 3.2% of the total osteotomies. BS occurred in 40% on the right side and 60% on the left. The mean age of patients was 30 years, with only one patient over 40 years. The male-to-female ratio was 1:1. The most affected deformity was Facial Pattern III, accounting for 60% of cases. The presence of a third molar was found in two cases. In two patients, there was an absence of at least one molar on the osteotomy side, and one BS case showed the presence of bicortical plates and screws from prior trauma surgery. Class A BS (fracture of the distal part of the proximal segment) was the most common, occurring in 60% of cases, followed by Class B (fracture of the distal part with involvement of the coronoid) in 30% of cases (Fig. 1,  Table 1).

Regarding the anatomical study of the affected side compared to the unaffected side, it was observed that in 40% of patients, the affected mandibular ramus was slightly thicker, 40% were similar, and 20% were narrower. In 50% of patients, the ramus presented decreased height compared to the other side, while 30% had similar height and 20% had greater height. Concerning the positioning of the lingula, 50% of the affected sides had the lingula in a more anterior position, 30% in a similar position, and 20% more posterior with the differences not being statistically significant. The measurements of anteroposterior length of the mandibular ramus and height of the crest showed balance between the sides ( Table 2).

**Figure 2:**
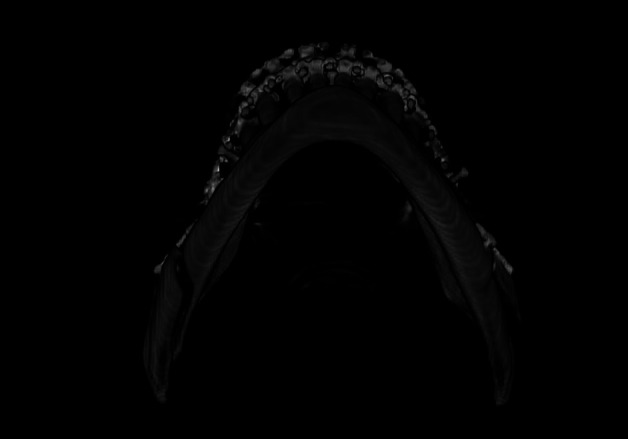
Example of technical deviation on the left side of the mandible, where the badsplit occurred. Note that the basilar osteotomy did not extend to the lingual side.

The technical evaluation of the osteotomy lines found that in 8 individuals (80%), there was a deviation from the opposite side or from what would be the recommended technique, with incomplete osteotomy at the basal level being the most identified alteration (60%) ( Table 3, Fig. 2).

## Discussion

Among the existing classifications of Bad Split (BS) (7), we utilized the Beukes *et al*. (2013) classification due to its practicality and representation of the patterns observed in our study. We found that 90% of BS cases affected the oral region of the proximal segment (Type A or B), with one-third involving the coronoid process (Type B), indicating that the oblique line region is the most susceptible to fractures.

We recorded a total incidence of 3.2% for unfavorable fractures, with an equal distribution between genders. There was no significant influence from the type of occlusion or age, which ranged from 18 to 43 years (mean age 30.1). These findings are consistent with other studies on the subject (8-12), suggesting that age, gender, and type of deformity are not risk factors.

The possibility of a third molar contributing to the occurrence of BS is often raised. Several studies (13,8,14,15) have indicated an increased intraoperative difficulty and a risk escalation of up to 9 times in the presence of these elements (15). However, systematic reviews on this topic (4,16) concluded that the scientific evidence is not robust enough to indicate a positive correlation.

In our study, we found that 20% presented with a third molar, 20% had edentulism, and 10% had previous osteosynthesis. This suggests that these factors may contribute to an increased risk of BS, as patients with these conditions are exceptions in our sample.

Additionally, numerous studies have analyzed mandibular anatomy through measurements to identify more susceptible patterns. It has been found that mandibles with less cancellous bone, where the distance from the canal to the cortex was shorter, were more prone to BS (17,18,19,5,12). It was also identified that, besides thickness, decreased height may increase the risk of BS (8,11).

Fujii *et al*. (2023) (22) further found that a high difference in thickness before and after entering the canal may favor BS. In contrast to previous studies, we did not find a difference in ramus thickness, but rather similar measurements between the sides when all BS cases were grouped together ( Table 2). However, when isolating only Class A fractures, the most prevalent at 60%, we found that 83.3% of cases had the lingula closer to the anterior edge than the control side, and 66% had a lower height of the bony crest. This suggests that anatomy may influence the type of BS when it occurs ( Table 4).

This data indicates that future research should separate anatomical analyses by BS pattern rather than grouping them together. The objective of the evaluation method (split mouth) was to investigate the reason for BS occurring on one side and not the other, observing the differences between them. Thus, technically assessing the executed osteotomy would also be crucial for this study.

In this evaluation, it was found that in 80% of BS cases, there was a “deviation” in the execution of the osteotomy, with incomplete osteotomy at the basal level being the most common deviation (60%) ( Table 4).

Several authors have highlighted the importance of the surgeon as a crucial factor. Studies involving a single surgeon have reported lower incidence rates (7,9,15) compared to studies from teaching hospitals (20,19). Thisunderscores the importance of the surgeon’s experience in reducing the number of cases, even in “prone” mandibles.

Efforts should always be made to avoid complications, and precautions with the osteotomy should include positioning the lingual cut just above the lingula, extending to the end of this structure; performing an oblique and vertical cut throughout its length down to the medullary area; and progressing the vertical cut at the base to the lingual (20,4,19,15,12). Furthermore, the cleavage should be approached very cautiously, starting from the basal and anterior regions before progressing posteriorly, always using fine chisels and applying light force. In case of resistance, the osteotomies and angles should be reassessed (20,4,19,15).

In cases of BS, one should aim to complete the separation of the segments as atraumatically as possible, typically through osteotomy in the proximal region of the distal segment. Intermediate segments should be kept adhered to the soft tissues and fixed to the main segments as much as possible (20,4,19,15).

## Conclusions

Factors such as the presence of third molars, edentulism, and fixation plates in the region appear to have a minor influence on the occurrence of Bad Splits. Mandibles with more anterior lingulae and lower heights of the alveolar crest are more susceptible to the occurrence of Class A Bad Split. Technical factors can strongly influence the occurrence of Bad Split, potentially being more determining than anatomical factors, placing the onus on the surgeon to execute the entire procedure as carefully as possible.

## Figures and Tables

**Table 1:** Description of the results in relation to the categorical variables and age of the patients in the sample.

Patient	Gender	Age	Classification of BadSplit	Type of deformity	Affected side	Local edentulism	Previous Local fixation	Third molar present
1	F	28	A	III	R	N	N	N
2	F	33	A	III	L	N	N	Y
3	F	30	A	III	L	N	N	N
4	M	34	A	III	R	Y	Y	N
5	M	24	A	III	L	N	N	N
6	M	33	A	II	L	N	N	N
7	M	18	C	II	L	N	N	N
8	M	28	B	II	R	N	N	Y
9	F	30	B	III	L	Y	N	N
10	F	43	B	II	R	N	N	N

**Table 2:** Description of the results of the mandibular anatomical analysis comparing the affected side and the unaffected side by BadSplit including all patients in the sample.

Variable	Group	N	Average ± DP	Median	Q 25 - 75	Min	Máx	p-value*
Thickness of the ascending ramus	With BS	10	8.46 ± 1.56	9.05	7.15 - 9.38	5.80	10.5	0.349
	Without BS	10	8.24 ± 1.53	8.25	7.78 - 9.28	5.30	10.5	
Height of the ascending ramus	With BS	10	44.59 ± 5.52	43.30	42.0 - 43.7	39.6	58.1	0.388
	Without BS	10	45.10 ± 5.56	44.00	42.9 - 45.0	36.5	57.5	
Lenght of the ascending ramus	With BS	10	27.40 ± 2.03	27.60	26.0 - 29.0	24.2	30.6	0.357
	Without BS	10	27.73 ± 1.80	27.70	26.4 - 28.6	25.1	30.4	
Lingula Location	With BS	10	14.66 ± 1.92	14.55	13.3 - 16.2	11.4	17.5	0.370
	Without BS	10	14.94 ± 1.60	14.95	14.3 - 15.9	12.5	18.0	
Alveolar Bone Crest Height	With BS	10	26.4 ± 3.83	26.3	23.2 - 27.8	22.1	33.0	0.799
	Without BS	10	26.9 ± 3.75	28.1	24.8 - 28.6	20.2	32.0	

**Table 3:** Description of the results of the mandibular anatomical analysis comparing the affected side and the unaffected side by BadSplit including only patients with type A badsplit.

Variable	Group	N	Average ± DP	Median	Q 25 - 75	Min	Máx	p-value*
Thickness of the ascending ramus	With BS	6	8,5 +- 1,8	9,25	7,4 – 9,38	5,8	10,5	0,68
	Without BS	6	8,62 +- 1,45	8,8	7,88 – 9,43	6,4	10,5	
Height of the ascending ramus	With BS	6	47 +- 6,01	43,7	43,38 – 48,38	4,3	58,1	1,41
	Without BS	6	47,52 +- 5,57	44,85	43,83 – 49,33	4,6	57,5	
Lenght of the ascending ramus	With BS	6	28,08 +- 1,86	28	27,55 – 29,05	25,1	30,6	0,72
	Without BS	6	28,22 +- 2,04	28,15	27,25 – 29,95	25,1	30,4	
Lingula Location	With BS	6	14,4 +- 2,1	14,35	13,35 – 15,43	11,4	17,5	0,04
	Without BS	6	15,23 +- 1,73	14,95	14,53 – 15,9	12,9	16,2	
Alveolar Bone Crest Height	With BS	6	25,77 +- 3,94	25,4	22,35 – 27,85	22,1	31,7	0,435
	Without BS	6	27,2 +- 3,26	28,3	25,35 – 28,55	22,3	31,3	

**Table 4:** Result of the tomographic analysis of the osteotomy performed.

Patient	Any deviation from the ideal technique	Which was identified?	Was the same defect identified on the other side?
1	Y	Incomplete osteotomy at the base	Y
2	Y	Incomplete osteotomy at the base and deviation of the vertical cut to the posterior	N
3	Y	Incomplete osteotomy at the base and deviation of the vertical cut to the posterior	N
4	N		Not applicable
5	N		Not applicable
6	Y	Incomplete osteotomy at the base	N
7	Y	Deviation of the vertical cut to the posterior region	N
8	Y	Very high horizontal osteotomy	N
9	Y	Incomplete osteotomy at the base	Y
10	Y	Incomplete osteotomy at the base	Y

## Data Availability

The datasets used and/or analyzed during the current study are available from the corresponding author.

## References

[1] Bockmann R, Meyns J, Dik E, Kessler P (2014). The modifications of the sagittal ramus split osteotomy: a literature review. Plast Reconstr Surg Glob Open.

[2] MacIntosh RB (1981). Experience with the Sagittal Osteotomy of the Mandibular Ramus: A 13-Year Review. J Maxillofac Surg.

[3] Peleg O, Mahmoud R, Shuster A, Arbel S, Manor Y, Ianculovici C (2021). Orthognathic surgery complications: The 10-year experience of a single center. J Cranio-Maxillofac Surg.

[4] Steenen SA, van Wijk AJ, Becking AG (2016). Bad splits in bilateral sagittal split osteotomy: systematic review and meta-analysis of reported risk factors. Int J Oral Maxillofac Surg.

[5] Fujii Y, Hatori A, Horiuchi M, Sugiyama-Tamura T, Hamada H, Sugisaki R (2023). Computed tomography evaluation of risk factors for an undesirable buccal split during sagittal split ramus osteotomy. PLoS One.

[6] Steenen SA, Becking AG (2016). Bad splits in bilateral sagittal split osteotomy: systematic review of fracture patterns. Int J Oral Maxillofac Surg.

[7] Beukes J, Reyneke JP, Becker PJ (2013). Variations in the anatomical dimensions of the mandibular ramus and the presence of third molars: its effect on the sagittal split ramus osteotomy. Int J Oral Maxillofac Surg.

[8] Wang T, Han JJ, Oh HK, Park HJ, Jung S, Park YJ (2016). Evaluation of Mandibular Anatomy Associated With Bad Splits in Sagittal Split Ramus Osteotomy of Mandible. J Craniofac Surg.

[9] Jiang N, Wang M, Bi R, Wu G, Zhu S, Liu Y (2021). Risk factors for bad splits during sagittal split ramus osteotomy: a retrospective study of 964 cases. Br J Oral Maxillofac Surg.

[10] Salzano G, Audino G, Friscia M, Vaira LA, Biglio A, Maglitto F (2022). Bad splits in bilateral sagittal split osteotomy: A retrospective comparative analysis of the use of different tools. J Craniomaxillofac Surg.

[11] Chandegra RK, Tsakalidis M, Pandya R, Stockton P (2023). The Unfavourable Split: a novel classification and an 11-year retrospective study looking at alternative methods for management of this well-known complication. Br J Oral Maxillofac Surg.

[12] Telha W, Abotaleb B, Zhang J, Bi R, Zhu S, Jiang N (2023). Correlation between mandibular anatomy and bad split occurrence during bilateral sagittal split osteotomy: a three-dimensional study. Clin Oral Investig.

[13] Verweij JP, Mensink G, Fiocco M, van Merkesteyn JPR (2014). Presence of mandibular third molars during bilateral sagittal split osteotomy increases the possibility of bad split but not the risk of other post-operative complications. J Cranio-Maxillofac Surg.

[14] Eshghpour M, Labafchi A, Samieirad S, Hosseini Abrishami M, Nodehi E, Rashid Javan A (2021). Does the Presence of Impacted Mandibular Third Molars Increase the Risk of Bad Split Incidence During Bilateral Sagittal Split Osteotomy?. World J Plast Surg.

[15] Chi H, Cai M (2022). A retrospective study of unfavorable fractures during sagittal split osteotomy: A single surgeon's experience. Chin J Plast Reconstr Surg.

[16] de Souza BB, da Silveira MLM, Dantas WRM, Almeida RAC, Germano AR (2023). Does the presence of third molars during sagittal split mandibular ramus osteotomy favour complications? Systematic review and meta-analysis. Int J Oral Maxillofac Surg.

[17] Muto T, Shigeo K, Yamamoto K, Kawakami J (2003). Computed tomography morphology of the mandibular ramus in prognathism: effect on the medial osteotomy of the sagittal split ramus osteotomy. J Oral Maxillofac Surg.

[18] Aarabi M, Tabrizi R, Hekmat M, Shahidi S, Puzesh A (2014). Relationship between mandibular anatomy and the occurrence of a bad split upon sagittal split osteotomy. J Oral Maxillofac Surg.

[19] Wang M, Li P, Zhang J, Sun Y, Zhang X, Jiang N (2022). Risk Factors Analysis for Different Types of Unfavorable Fracture Patterns During Sagittal Split Ramus Osteotomy: A Retrospective Study of 2008 Sides. Aesthetic Plast Surg.

[20] Falter B, Schepers S, Vrielinck L, Lambrichts I, Thijs H, Politis C (2010). Occurrence of bad splits during sagittal split osteotomy. Oral Surg Oral Med Oral Pathol Oral Radiol Endod.

[21] Gateno J, Ferraz F, Alfi D (2015). Bad split: anatomic or technical problem?. J Oral Maxillofac Surg.

[22] Fujii Y, Hatori A, Horiuchi M, Sugiyama-Tamura T, Hamada H, Sugisaki R (2023). Computed tomography evaluation of risk factors for an undesirable buccal split during sagittal split ramus osteotomy. PLoS One.

